# Partial motor status epilepticus as a clinical manifestation of carotid stenosis

**DOI:** 10.1186/1755-7682-3-18

**Published:** 2010-09-06

**Authors:** Renée Ribacoba, Manuel Menéndez-González, Sergio Calleja, Javier Salas-Puig, Vanessa de la Vega

**Affiliations:** 1 Unit of Neurology, Hospital Alvarez Buylla, Mieres, Asturias, Spain; 2 Service of Neurology, Hospital Universitario Central de Asturias, Oviedo, Astuias, Spain

## Abstract

Limb shaking (LS) is often confused with focal motor seizures. Distinguishing between both is crucial, because LS may represent an indicator of severe carotid occlusive disease and patients are at high risk of stroke. We report the case of a patient with occlusive carotid stenosis without definite stroke who develops partial motor status epilepticus (SE). Clinical, neuroimaging and electroencephalographic findings are provided. We conclude that focal motor seizures should be distinguished from LS based on clinical and electroencephalographic findings.

## Introduction

Since Miller Fischer described limb shaking (LS) in 1962 as a rare clinical manifestation of severe stenosis carotid in humans with transitory ischemic attack (TIA) [1] this diagnosis has been reported regularly [2]. However in rat models of middle cerebral artery occlusion with cerebral infarction periodic lateralized epileptiform discharges (PLEDs) occurred ipsilateral to the lesion within the first 72-hour period (ischemic and reperfusion) in most of the animals tested [3].

LS is often confused with focal motor seizures. Distinguishing between both is crucial, because LS may represent an indicator of severe carotid occlusive disease and patients are at high risk of stroke [4]. The clinical features of LS are rhythmic or arrhythmic involuntary hyperkinesias affecting the hand, arm, leg, hand-arm, or hand-arm-leg unilaterally. The face muscles are always spared and tonic contractions, tonic-clonic jerking "jacksonian march" or choreathetotic movement have never been observed. Upper limb are more evidently affected. During attacks conscience is preserved. The frequency is variable from isolated episodes to several episodes a day, lasting for seconds or minutes.

In the following report we present a case of partial motor status epilepticus with preocclusive carotid stenosis without stroke that responded to treatment with antiepileptic drugs.

## Case report

The patient is a 74-years-old right-handed man with a history of hypertension and emphysema. While working in his garden he felt dizziness. He developed dysarthria and right hemiparesis. He was admitted at our hospital and 75 minutes later he suffered the first seizure: his eyes turned to the right, the right arm rose up and developed myoclonic jerks in right arm, leg and face during one minute. His consciousness was impaired during the attack. Two hours later, he suffered two right partial motor seizures secondarily generalised. He was put on treatment with diazepan intravenous and valproic acid. On admission the clinical exam showed right hemiplegia, motor aphasia and was semiunconscious. His brain CT scan and cerebrospinal fluid analysis were normal. In the following hours he developed a status epilepticus (SE) defined by regular or irregular myoclonic jerk involving the right side of the face and distal upper limb with anartria. He stayed for 25 days in the Intensive Care Unit with assisted ventilation and intravenous treatment with up to four antiepileptic drugs. Currently the patient suffers a dysphasic aftermath. He is on antiplatelet treatment and two antiepileptic drugs (levetiracetam 1500 mg per day and phenitoin 300 mg per day) and has not suffered more seizures.

## Results

Several ictal video-EEGs were performed. A Video-EEG performed 12 hours after admission showed sharp wave or tryphasic activity on the left frontotemporal area with spontaneous clonic twitching in right side of his face. Another one (additional file 1) registered a partial motor secondarily generalized seizure lasting 144 seconds; intravenous Clonazepam stopped the clinical episode but left frontal polyspikes continued. The next EEGs showed left focal frontal sharp waves (additional file 1).

**Figure 1:**
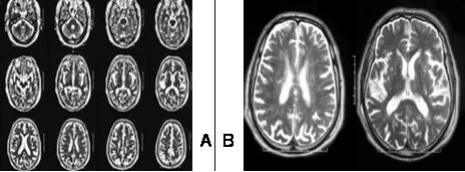
**Two independent MRI showed leukoaraiosis in T2 weighted images while diffusion images were normal**. Never acute ischemic lesion was observed. a) MRI performed in the forth day of evolution. b) Detail of the MRI performed in the 76^th ^day.

Two MRIs (fig 1) obtained at days 4 and 76 days showed leukoaraiosis in T2 weighted images while diffusion images were normal.

**Figure 2:**
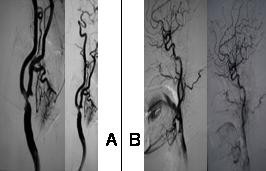
a) A carotid angiography confirmed a stenosis of 90% in left ICA; b) The post-stent angiography was normal.

Evaluation of carotid Doppler and carotid bifurcation with MRI angiographic techniques showed a calcified atherosclerotic plaque causing a high grade left stenosis in the internal carotid artery. A carotid angiography confirmed a stenosis of 90%(fig 2a). A stent was placed in the left Internal Carotid Artery (ICA) and the control angiography was normal (fig 2b).

**Figure 3:**
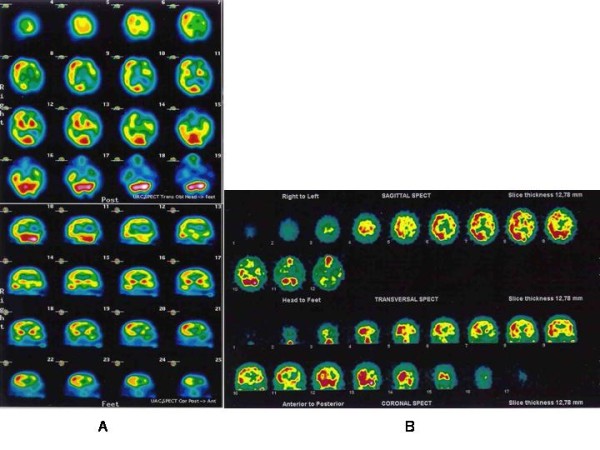
a) SPECT scan eight days later showed decreased perfusion on the posterior portion of the left parietal lobe and inferior temporal lobe area; b) Brain SPECT seven months later revealed severe hypoperfusion in the left hemisphere.

A brain SPECT scan eight days later (fig 3a) revealed decreased perfusion on the posterior portion of the left parietal lobe and inferior temporal lobe area. Another brain SPECT seven months later (fig. 3b) showed severe hypoperfusion in the left hemisphere in spite of the fact that circulation had been restablished.

## Discussion

Traditionally LS is a phenomenon associated to hypoperfusion in an ischemic territory (typically boundary territory with worse perfusion) linked to exhausted vasomotor reactivity [5]. Typically the phenomenon appears when the patient stands up or seats down and disappears in lied down position [6]. Usually EEG recording is normal and a simultaneous delta rhythm was found in the exceptional cases with ictal video-EEG, suggesting decreased perfusion [7].

Han et al. [7] analysed three brain SPECTs in an isolated case with focal stenosis of the anterior cerebral artery that developed recurrent shaking movement of a lower limb; 1. baseline SPECT: showed no perfusion defects, 2. SPECT after acetazolamide: the perfusion decreased in the medial aspect of the right frontal lobe, suggesting a lack of an autoregulatory reserve and 3. the ictal SPECT showed increased perfusion in the same region. Authors explain this finding suggests that the hyperkinetic movements may be associated with a hyperfunction of cortical neurons rather than a direct cortical suppression because cerebral blood flow is coupled to neuronal activity in most physiologic and pathologic conditions. According to this, there has been shown that neuronal hyperexcitability is found in ischemic tissue through the accumulation of free radicals and excitatory aminoacids [8]. In our patient, only two brain SPECT could be performed. The first, was performed one hundred days to status showed regional hypoperfusion. The second brain SPECT seven months later revealed an extensive hypopefusion in all left hemisphere. Unfortunately, we did not obtained ictal SPECT during status looking for characteristic focal hyperperfusion.

In this case, there was not acute ischemic damage on MRI, therefore we can not consider that a stroke is the cause of the epileptic phenomena. A difficult question to assess is the importance of leukoaraiosis in the development of the status epilepticus, but previous studies [9] reported that it can be a factor that eases the epileptic phenomena in a exhaust tissue.

Another question in this case are the definitive postepileptic functional deficits. These deficits are usually transient [10] and normalization of the cortical functions often appears after a few minutes or hours. An exception is in the hemiconvulsion-hemiplegia-epilepsy-syndrome [11] where prolonged hemiconvulsive clonic status is followed by permanent hemiplegia. The patophysiology of this syndrome is not fully understood, but cellular damage and neuronal death secondary to repetitive seizures have been considered. Prolonged postictal deficit have been described previously without leukoaraiosis or ipsilateral occlusive carotid disease [12].

Shimoda et al. [13] published a case of occlusive carotid syndrome and seizures that responded to anticonvulsant therapy, but they not performed concomitant EEG recording and the possibility of focal symptomatic epilepsy due to stroke is present. Then, in our knowledge this is the first case with occlusive stenosis of the ICA without definite stroke showing an epileptiform activity on EEG and response to antiepileptic drugs.

## Conclusions

We conclude that focal motor seizures should be distinguished from LS based on clinical and electroencephalographic findings. Though both might represent an alarm sign related to an occlusive carotid stenosis as showed in this case, generally the clinical meaning and management differ.

## Competing interests

The authors declare that they have no competing interests.

## Authors' contributions

RR, MM, SC and VV were involved in the direct care of this patient. All authors wrote, read and approved the final manuscript.

## Consent

Written informed consent was obtained from the patient for publication of this case report and any accompanying images. A copy of the written consent is available for review by the Editor-in-Chief of this journal.

## Supplementary Material

Additional file 1 **Ictal video-EEGs**. 1. EEG recording #1 showing one clinical right motor partial secondarily generalized seizure. Unfortunately, we no obtained permission for publishing the video. On the fourteenth and fifteenth channel an artefact resulting from poor contact is observed. On slow background observing frontal parasagittal generation with maximum negativity at F3. In following screens, the focus extends to parasagittal and temporal areas. Later, rhythmic polyspikes-wave extends to both hemispheres. Finally, when seizure finishes, polyspikes-slow waves for several seconds (15 mm = 1 sec; A = 50 mv). 2. EEG recording #2 at 28 minutes. IV Clonazepam stopped the clinical episode but left frontal pseudoperiodic activity continued (15 mm = 1 sec; A = 50 mv). 3. EEG recording #3 at 30 minutes showing similar findings to previous (15 mm = 1 sec; A = 50 mv). Click here for file
