# Supplementary material for: Partial motor status epilepticus as a clinical manifestation of carotid stenosis
Source: Int Arch Med. 2010 Sep 6;3:18. doi: 10.1186/1755-7682-3-18 (PMC2942812; doi:10.1186/1755-7682-3-18)
Supplement: Additional file 1 — Ictal video-EEGs. 1. EEG recording #1 showing one clinical right motor partial secondarily generalized seizure. Unfortunately, we no obtained permission for publishing the video. On the fourteenth and fifteenth channel an artefact resulting from poor contact is observed. On slow background observing frontal parasagittal generation with maximum negativity at F3. In following screens, the focus extends to parasagittal and temporal areas. Later, rhythmic polyspikes-wave extends to both hemispheres. Finally, when seizure finishes, polyspikes-slow waves for several seconds (15 mm = 1 sec; A = 50 mv). 2. EEG recording #2 at 28 minutes. IV Clonazepam stopped the clinical episode but left frontal pseudoperiodic activity continued (15 mm = 1 sec; A = 50 mv). 3. EEG recording #3 at 30 minutes showing similar findings to previous (15 mm = 1 sec; A = 50 mv). [file 1755-7682-3-18-S1.PDF]

Additional File: EEG recordings for "Partial motor status epilepticus as a clinical manifestation of carotid stenosis".

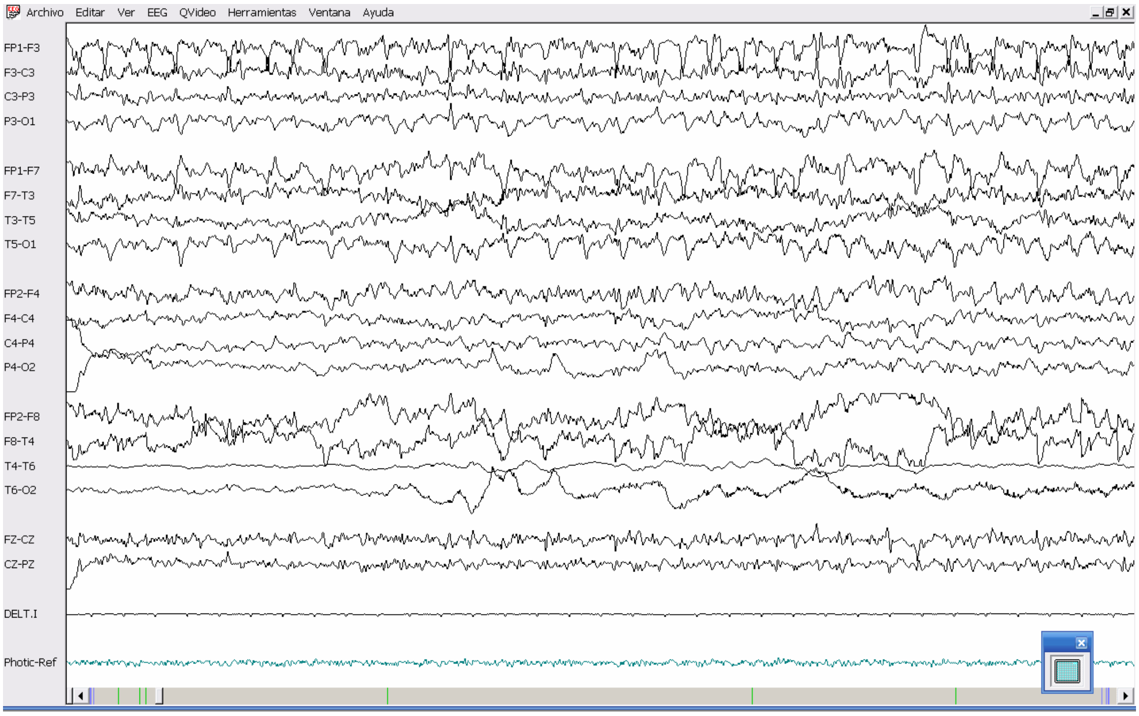

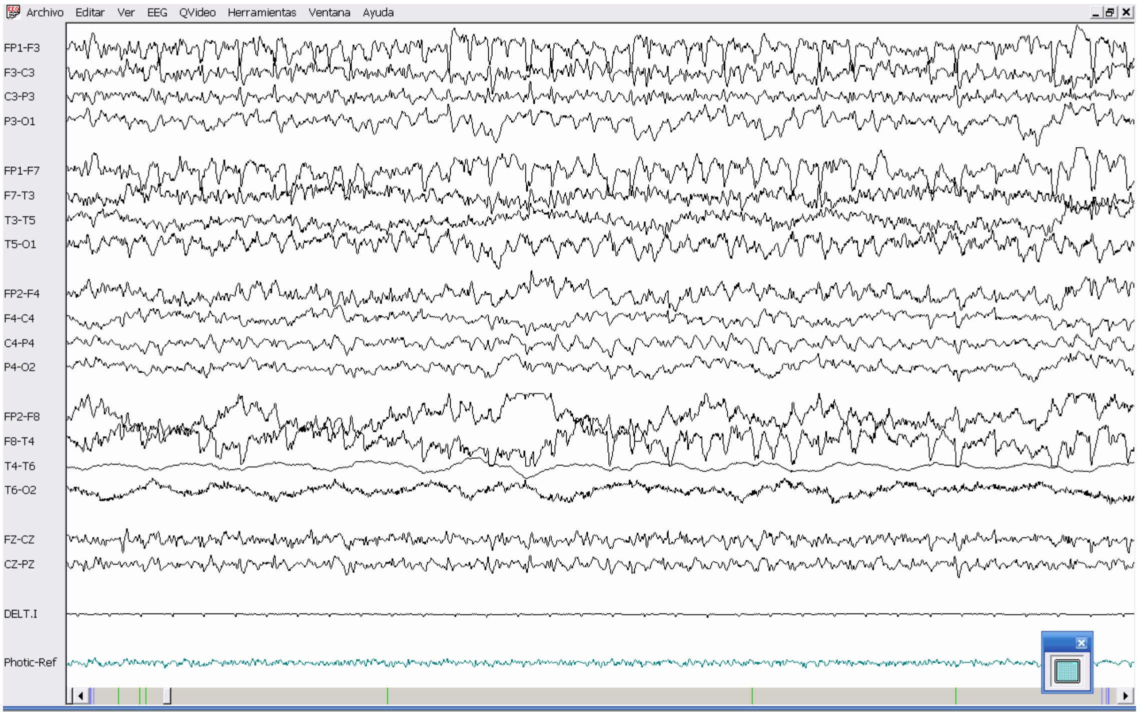

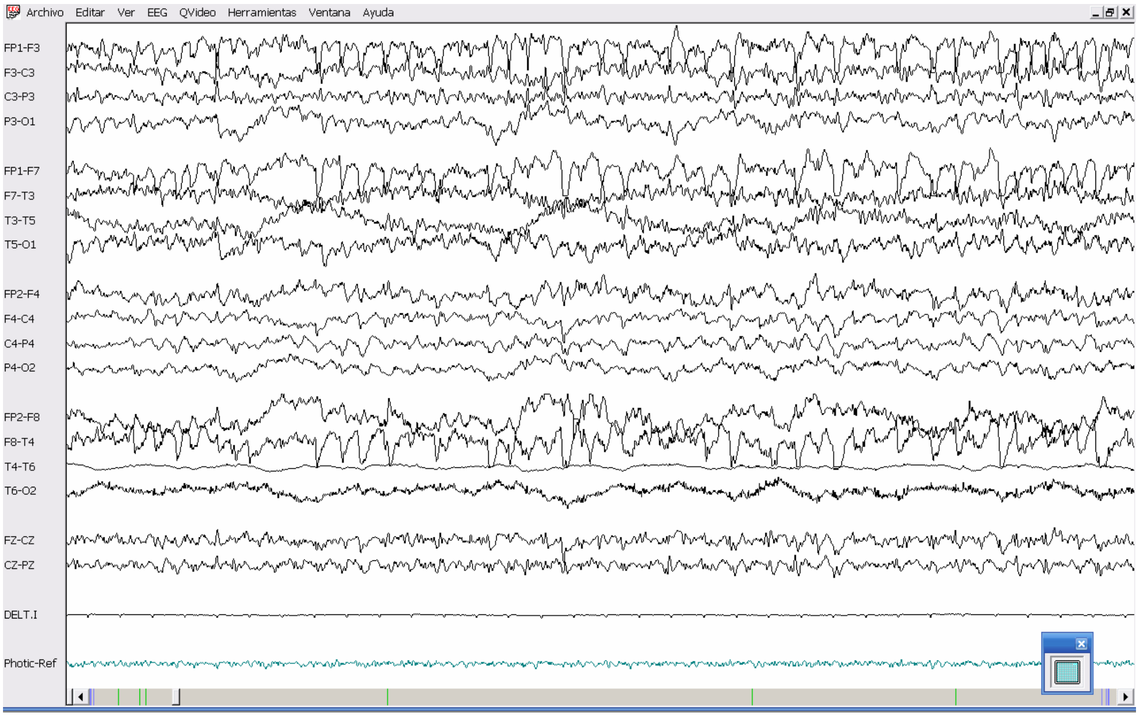
Archivo
Editar
Ver
EEG
QVideo
Herramientas
Ventana
Ayuda
FP1-F3
F3-C3
C3-P3
P3-O1
FP1-F7
F7-T3
T3-T5
T5-O1
FP2-F4
F4-C4
C4-P4
P4-O2
FP2-F8
F8-T4
T4-T6
T6-O2
FZ-CZ
CZ-PZ
DELT.I
Photic-Ref

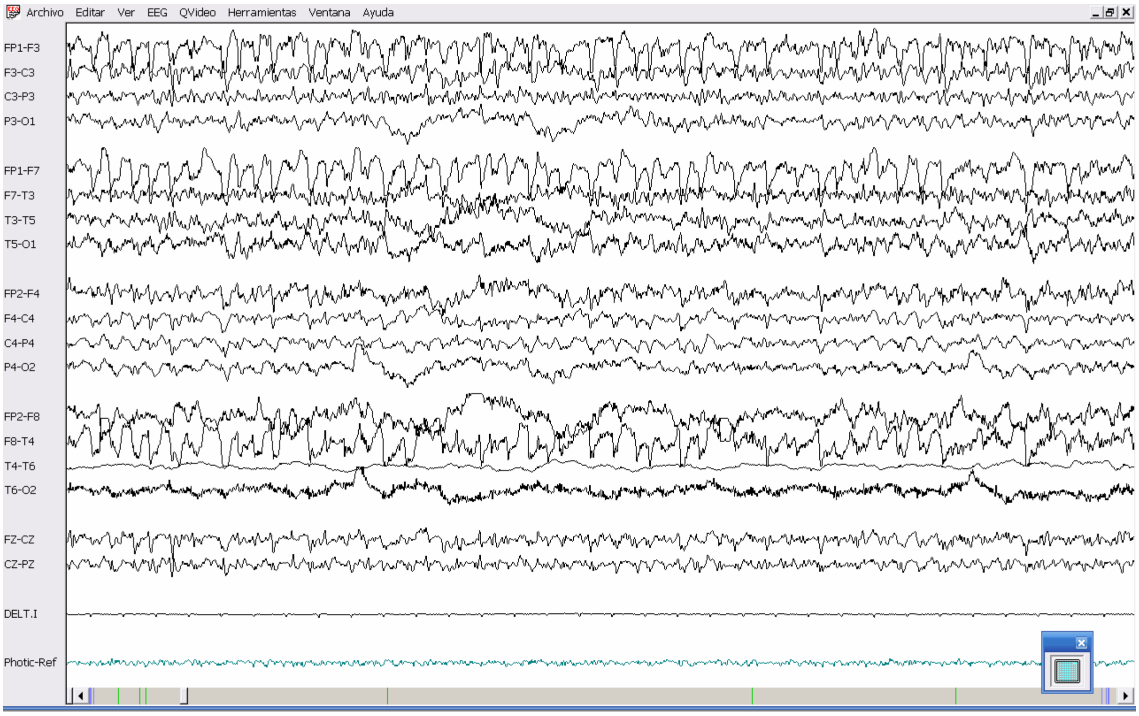
Archivo
Editar
Ver
EEG
QVideo
Herramientas
Ventana
Ayuda
FP1-F3
F3-C3
C3-P3
P3-O1
FP1-F7
F7-T3
T3-T5
T5-O1
FP2-F4
F4-C4
C4-P4
P4-O2
FP2-F8
F8-T4
T4-T6
T6-O2
FZ-CZ
CZ-PZ
DELT.I
Photic-Ref

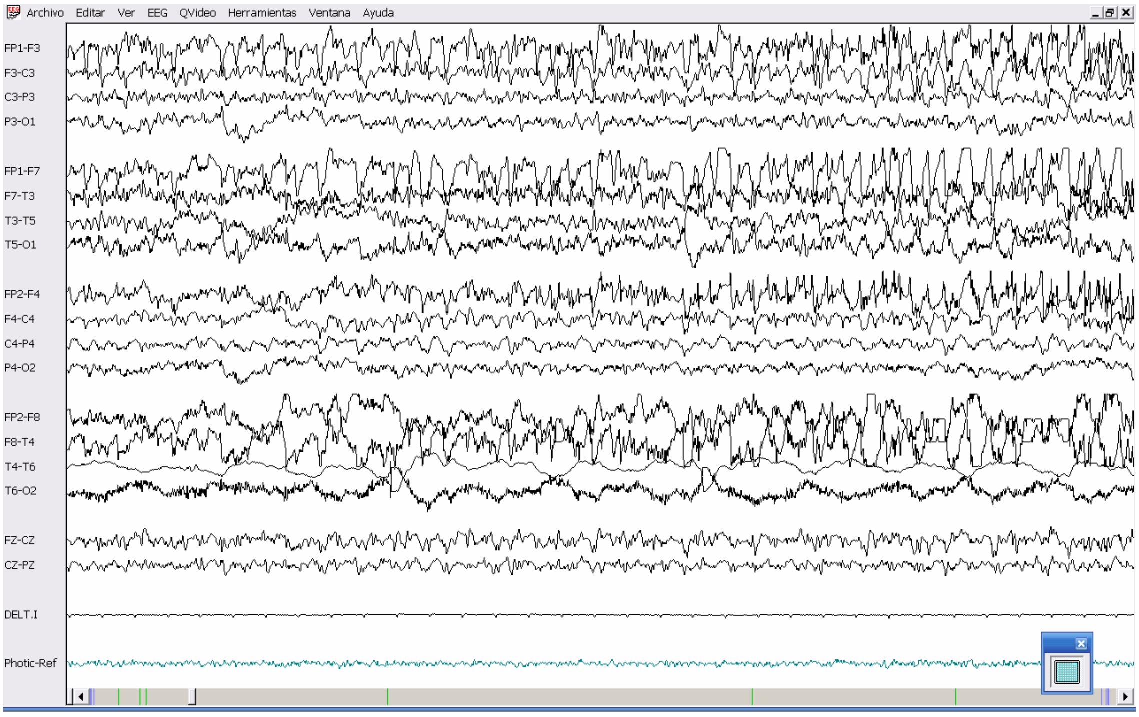
Archivo
Editar
Ver
EEG
QVideo
Herramientas
Ventana
Ayuda
FP1-F3
F3-C3
C3-P3
P3-O1
FP1-F7
F7-T3
T3-T5
T5-O1
FP2-F4
F4-C4
C4-P4
P4-O2
FP2-F8
F8-T4
T4-T6
T6-O2
FZ-CZ
CZ-PZ
DELT.I
Photic-Ref

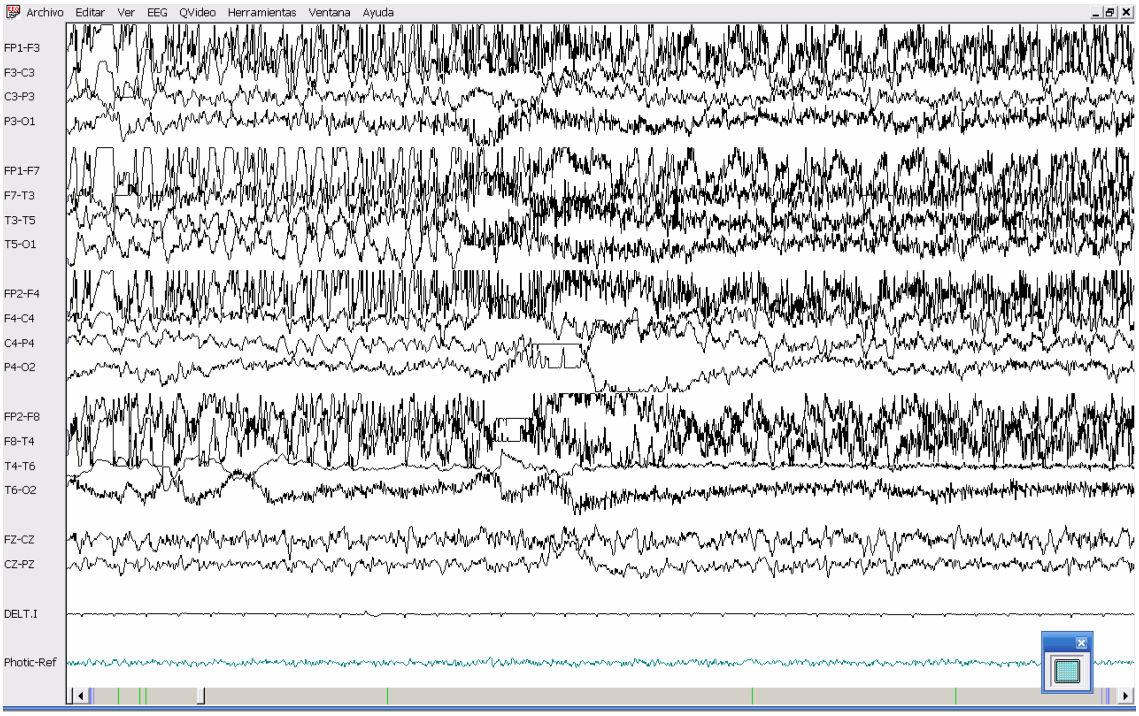
Archivo
Editar
Ver
EEG
QVideo
Herramientas
Ventana
Ayuda
FP1-F3
F3-C3
C3-P3
P3-O1
FP1-F7
F7-T3
T3-T5
T5-O1
FP2-F4
F4-C4
C4-P4
P4-O2
FP2-F8
F8-T4
T4-T6
T6-O2
FZ-CZ
CZ-PZ
DELT.I
Photic-Ref

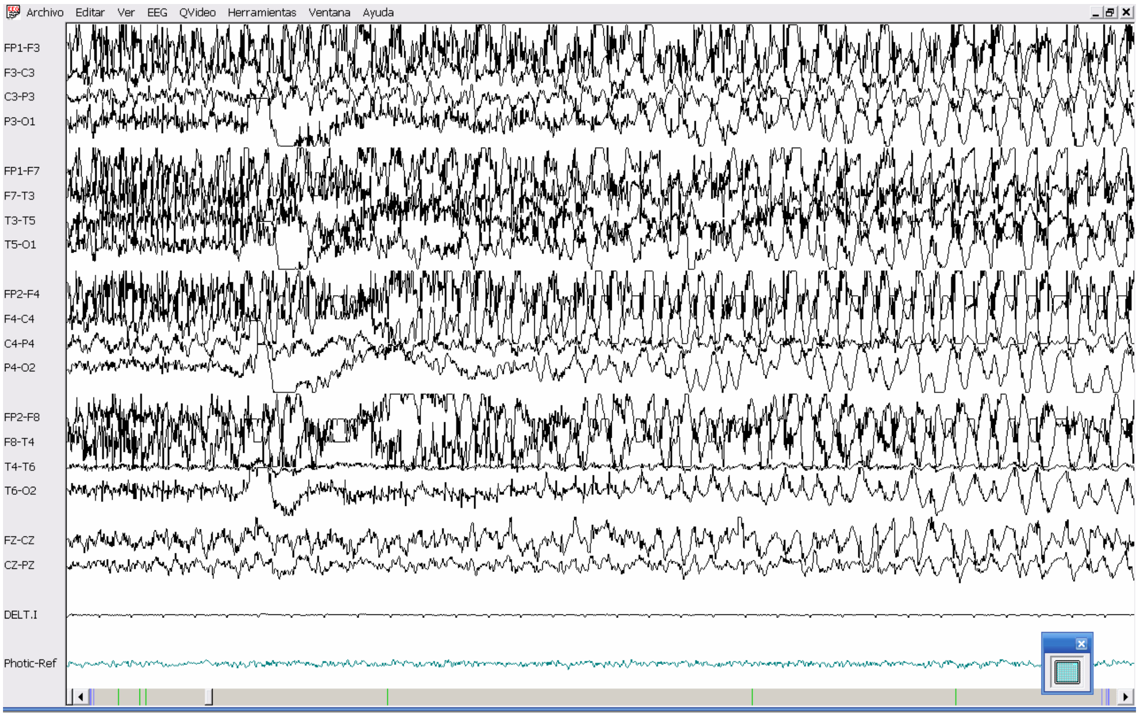
Archivo Editar Ver EEG QVideo Herramientas Ventana Ayuda
FP1-F3
F3-C3
C3-P3
P3-O1
FP1-F7
F7-T3
T3-T5
T5-O1
FP2-F4
F4-C4
C4-P4
P4-O2
FP2-F8
F8-T4
T4-T6
T6-O2
FZ-CZ
CZ-PZ
DELT.I
Photic-Ref

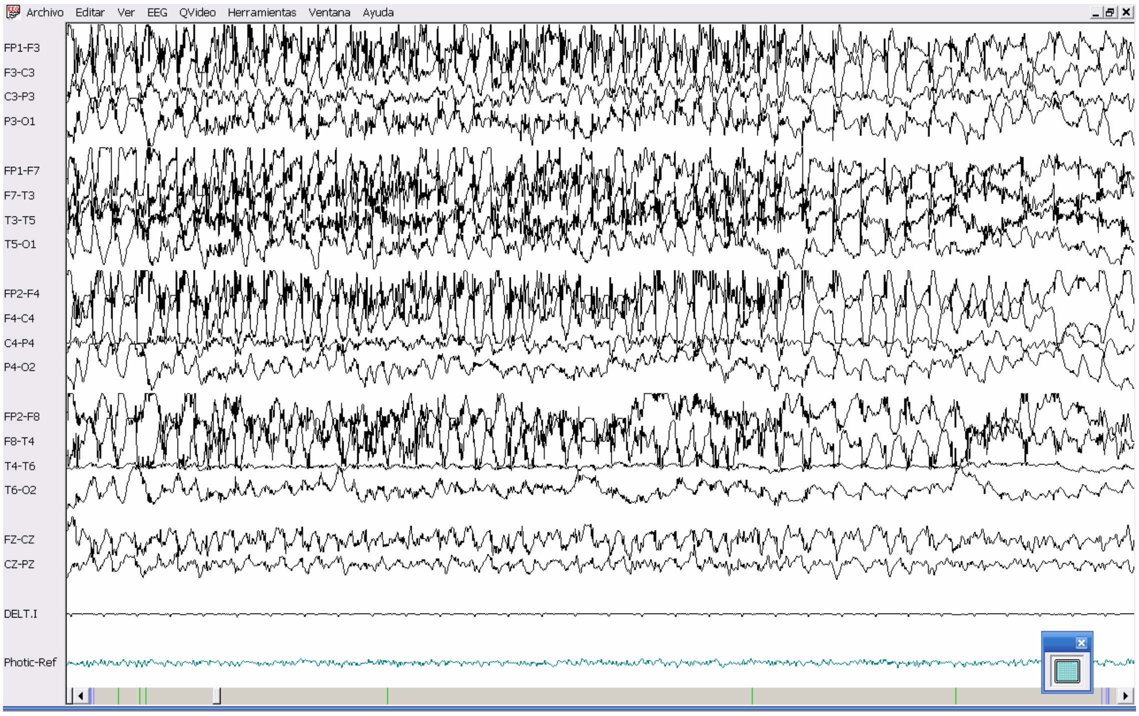
Archivo Editar Ver EEG QVideo Herramientas Ventana Ayuda
FP1-F3
F3-C3
C3-P3
P3-O1
FP1-F7
F7-T3
T3-T5
T5-O1
FP2-F4
F4-C4
C4-P4
P4-O2
FP2-F8
F8-T4
T4-T6
T6-O2
FZ-CZ
CZ-PZ
DELT.I
Photic-Ref

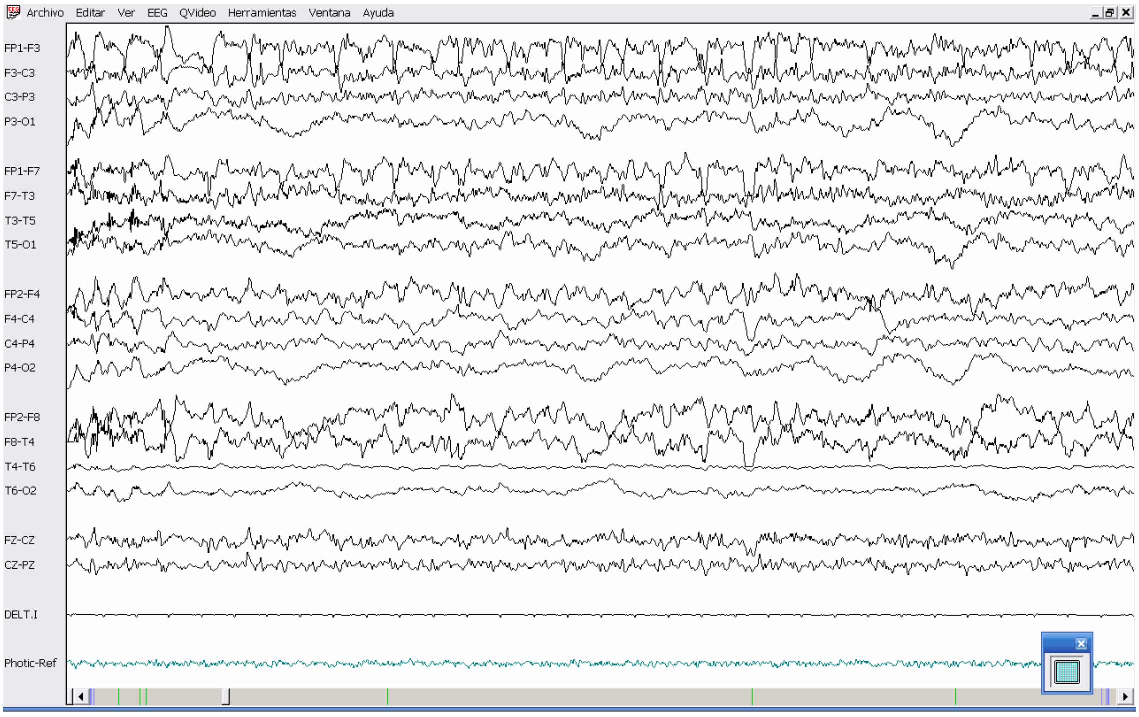

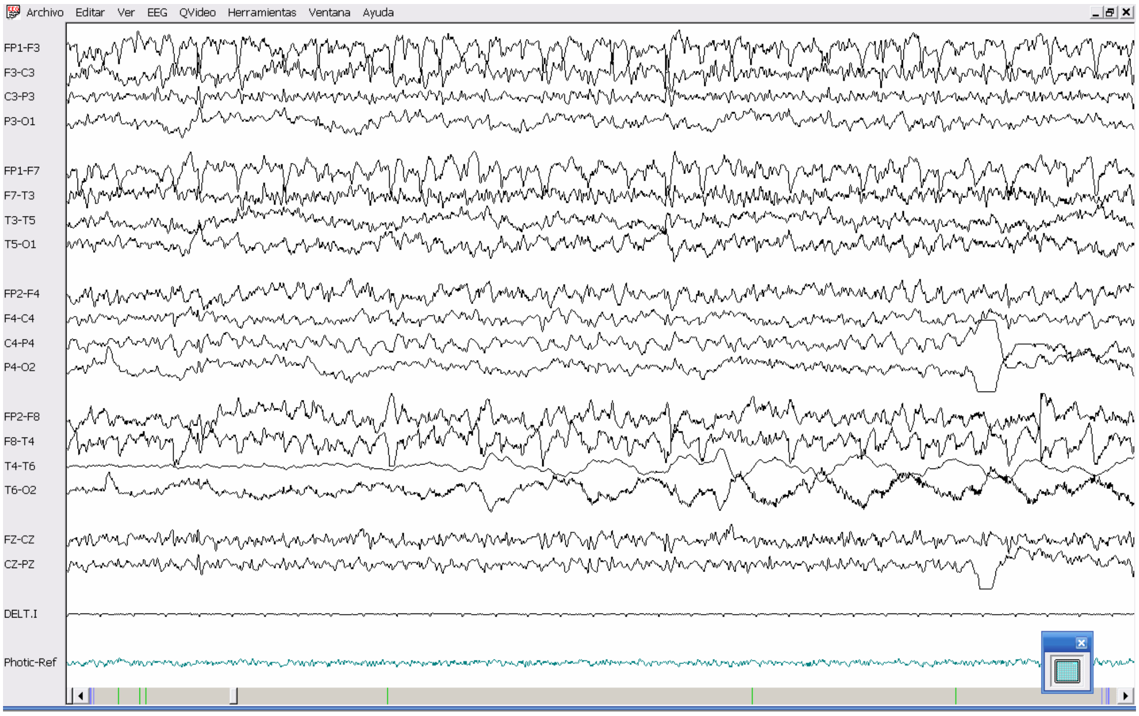

1st EEG recording showing one clinical right motor partial secondarily generalized seizure. Unfortunately, we no obtained licence for publish video. On the fourteenth and fiveteenth channel an artifact resulting from poor contact is observed. On slow background observing frontal parasaggittal generation with maximum negativity at F3. In following, field extends to parasaggital and temporal areas. Later, rhythmic polyspikes-wave extends in both hemispheres. Finally, when seizure finish, polyspikes-slow wave belong during several seconds and then,

a recording similar at he first, has been obtained. (15 mm = 1 sec; A = 50 mv)

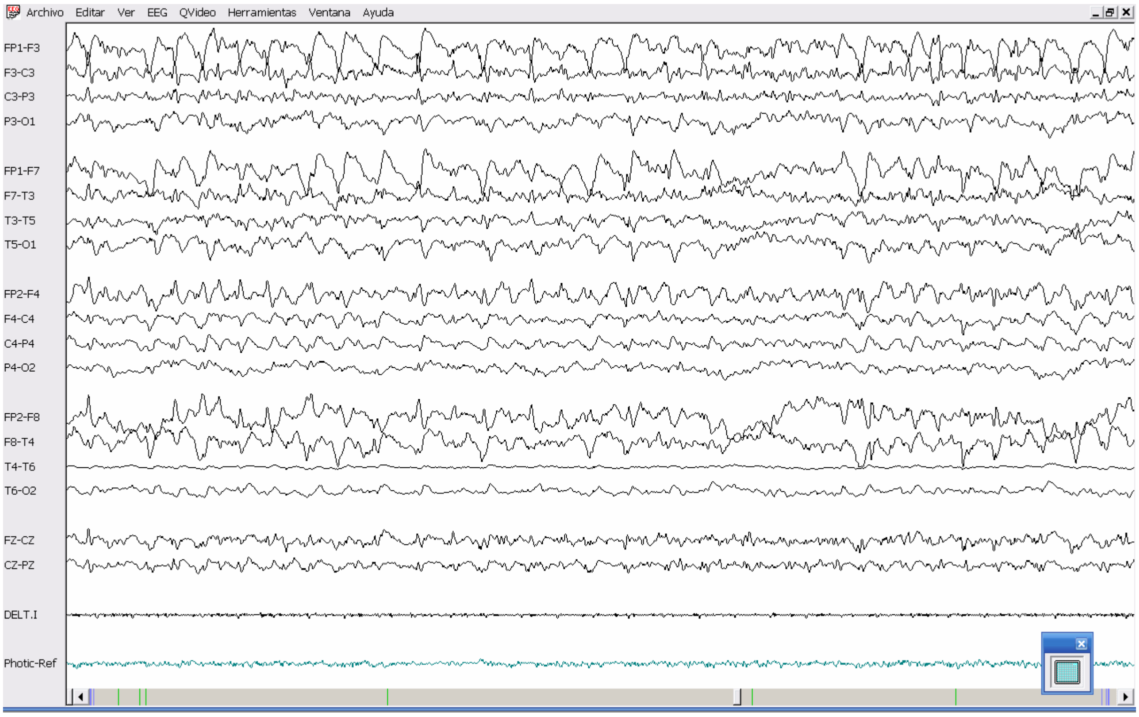
Archivo Editar Ver EEG QVideo Herramientas Ventana Ayuda
FP1-F3
F3-C3
C3-P3
P3-O1
FP1-F7
F7-T3
T3-T5
T5-O1
FP2-F4
F4-C4
C4-P4
P4-O2
FP2-F8
F8-T4
T4-T6
T6-O2
FZ-CZ
CZ-PZ
DELT.I
Photic-Ref

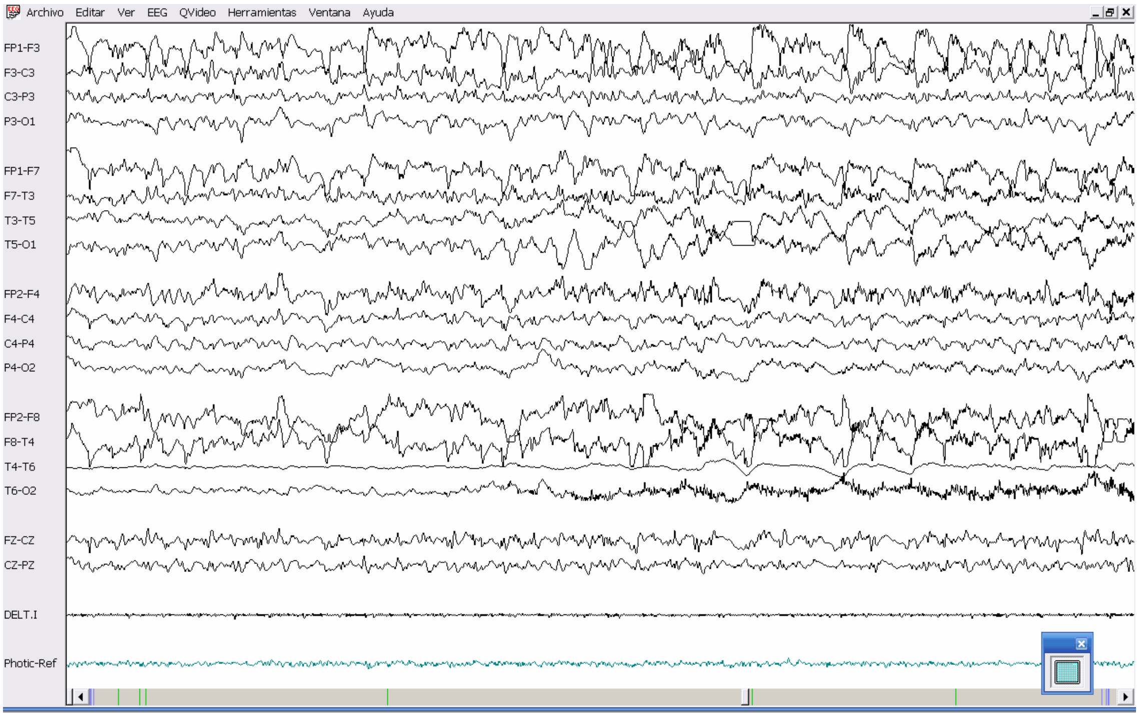
Archivo Editar Ver EEG QVideo Herramientas Ventana Ayuda
FP1-F3
F3-C3
C3-P3
P3-O1
FP1-F7
F7-T3
T3-T5
T5-O1
FP2-F4
F4-C4
C4-P4
P4-O2
FP2-F8
F8-T4
T4-T6
T6-O2
FZ-CZ
CZ-PZ
DELT.I
Photic-Ref

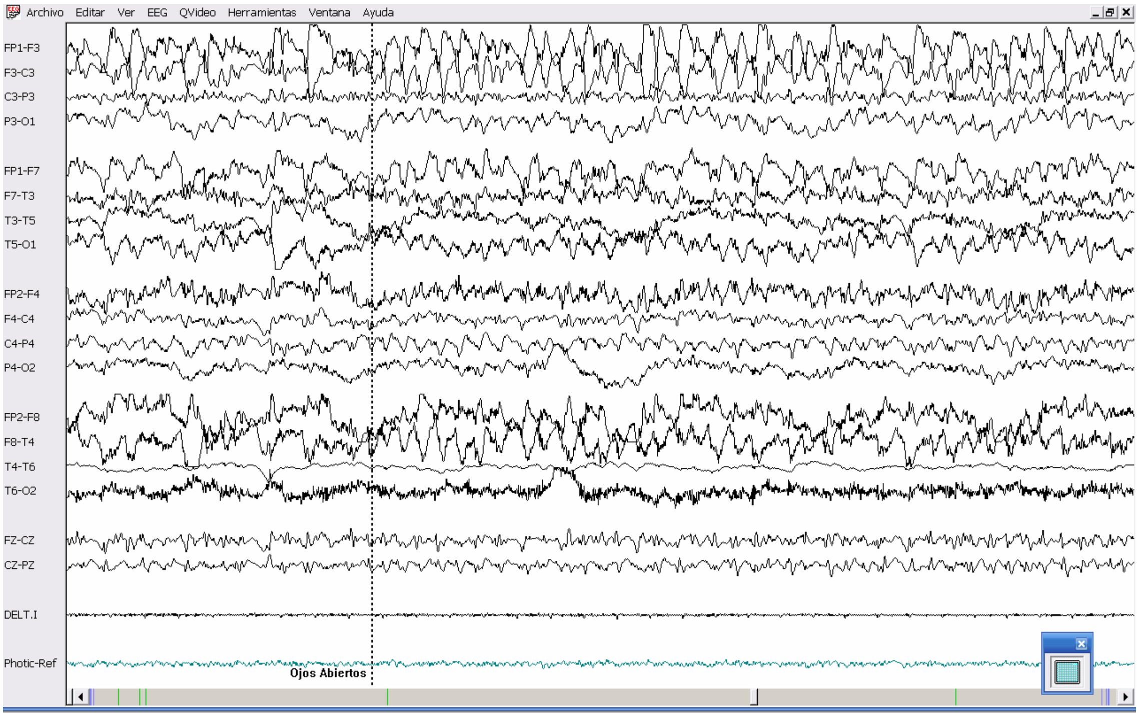
Archivo Editar Ver EEG QVideo Herramientas Ventana Ayuda
FP1-F3
F3-C3
C3-P3
P3-O1
FP1-F7
F7-T3
T3-T5
T5-O1
FP2-F4
F4-C4
C4-P4
P4-O2
FP2-F8
F8-T4
T4-T6
T6-O2
FZ-CZ
CZ-PZ
DELT.I
Photic-Ref
Ojos Abiertos

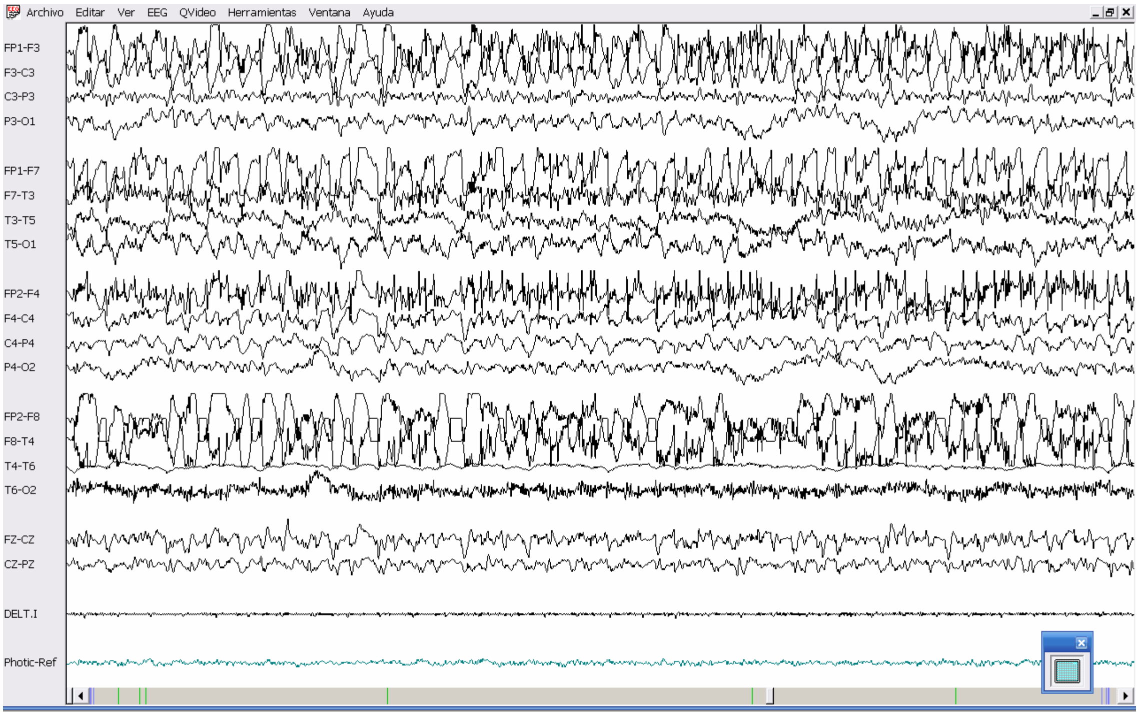
Archivo Editar Ver EEG QVideo Herramientas Ventana Ayuda
FP1-F3
F3-C3
C3-P3
P3-O1
FP1-F7
F7-T3
T3-T5
T5-O1
FP2-F4
F4-C4
C4-P4
P4-O2
FP2-F8
F8-T4
T4-T6
T6-O2
FZ-CZ
CZ-PZ
DELT.I
Photic-Ref

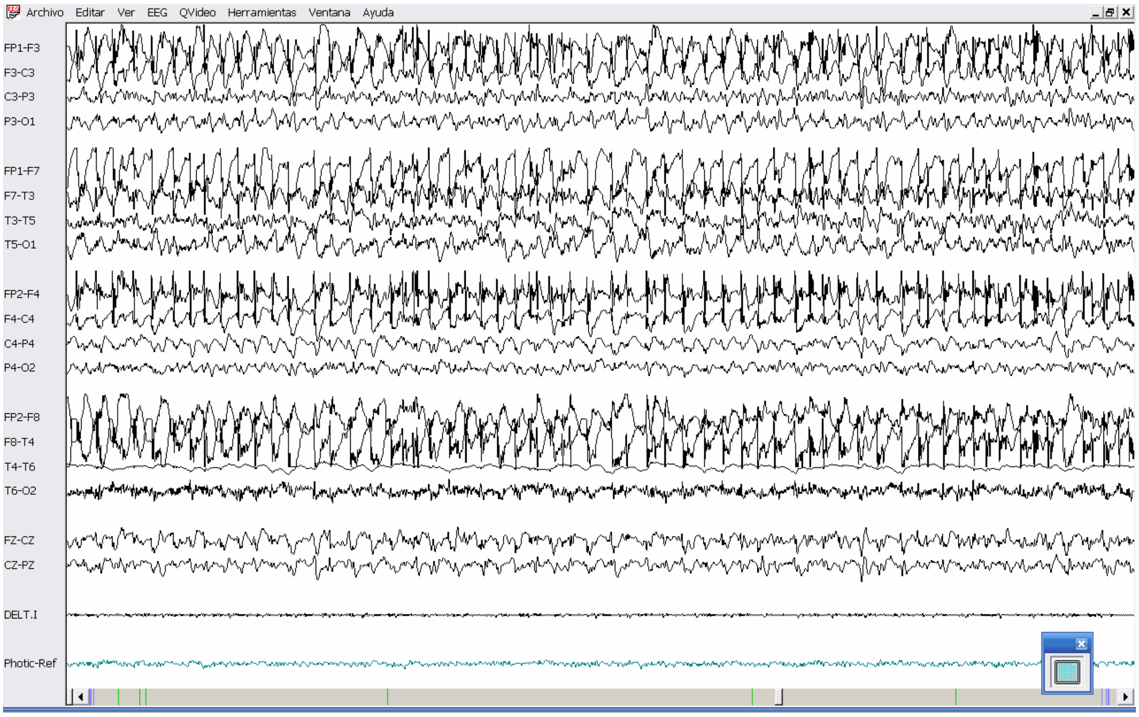
Archivo Editar Ver EEG QVideo Herramientas Ventana Ayuda
FP1-F3
F3-C3
C3-P3
P3-O1
FP1-F7
F7-T3
T3-T5
T5-O1
FP2-F4
F4-C4
C4-P4
P4-O2
FP2-F8
F8-T4
T4-T6
T6-O2
FZ-CZ
CZ-PZ
DELT.I
Photic-Ref

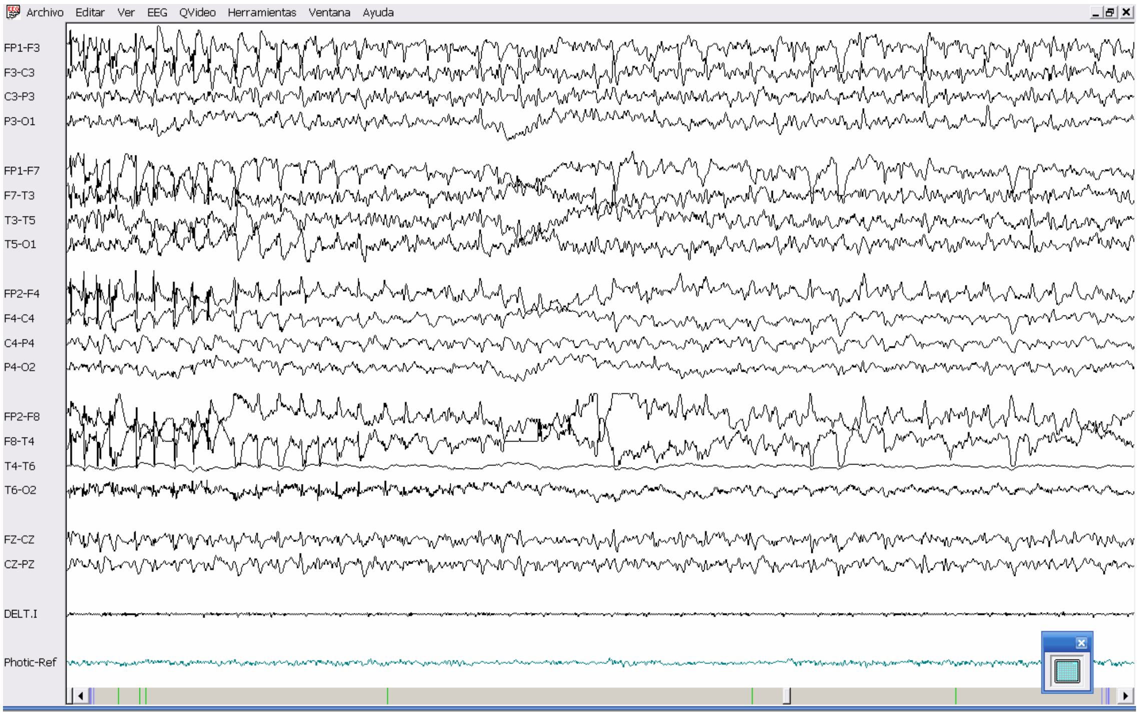
Archivo Editar Ver EEG QVideo Herramientas Ventana Ayuda
FP1-F3
F3-C3
C3-P3
P3-O1
FP1-F7
F7-T3
T3-T5
T5-O1
FP2-F4
F4-C4
C4-P4
P4-O2
FP2-F8
F8-T4
T4-T6
T6-O2
FZ-CZ
CZ-PZ
DELT.I
Photic-Ref

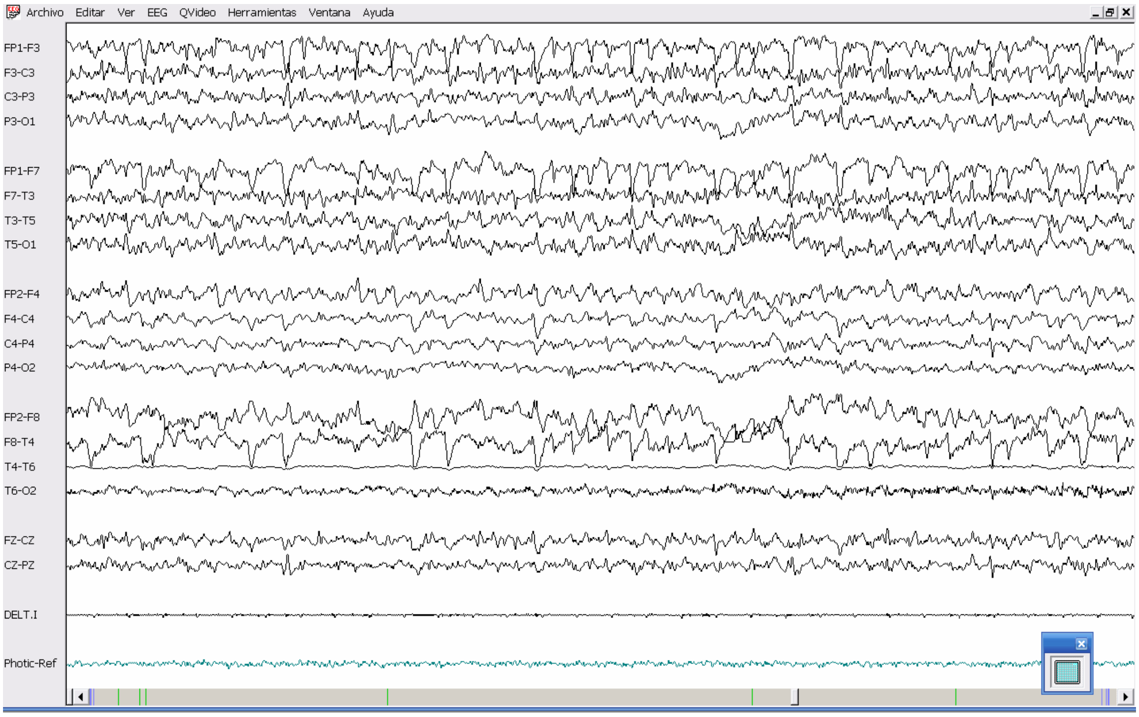

2nd EEG recording at 28 minutes. IV Clonazepam stopped the clinical episode but left frontal pseudoperiodic activity continued. Three more seizures were recorded. (15 mm = 1 sec; A = 50 mv)

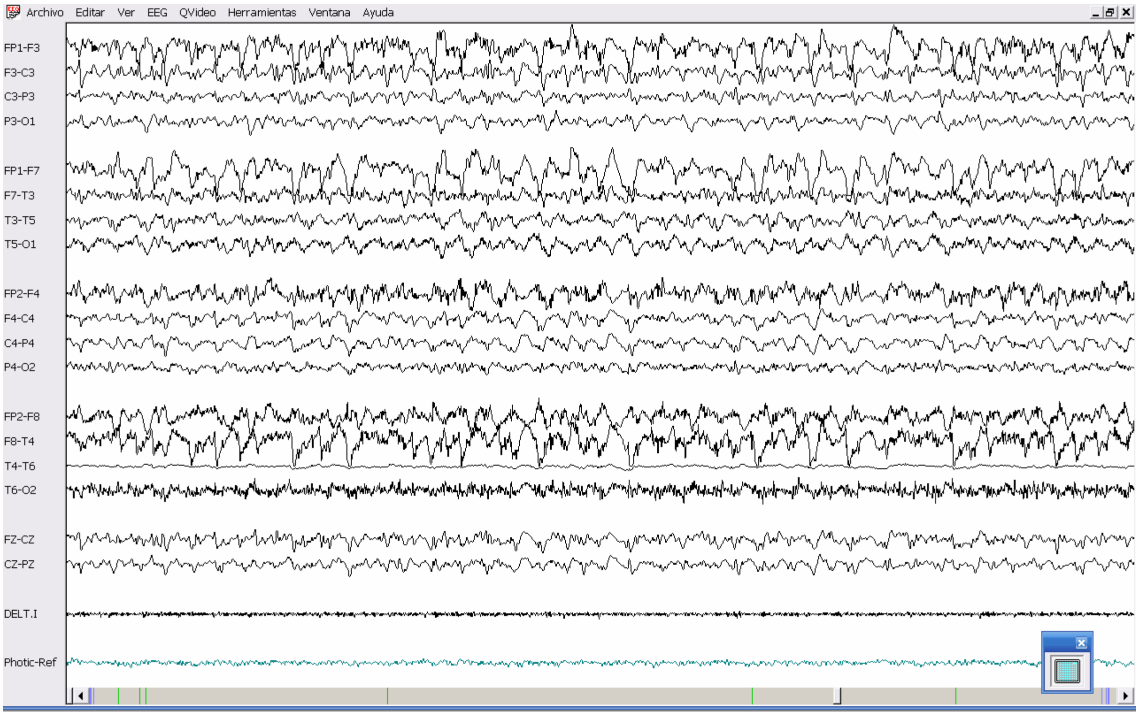
Archivo Editar Ver EEG QVideo Herramientas Ventana Ayuda
FP1-F3
F3-C3
C3-P3
P3-O1
FP1-F7
F7-T3
T3-T5
T5-O1
FP2-F4
F4-C4
C4-P4
P4-O2
FP2-F8
F8-T4
T4-T6
T6-O2
FZ-CZ
CZ-PZ
DELT.I
Photic-Ref

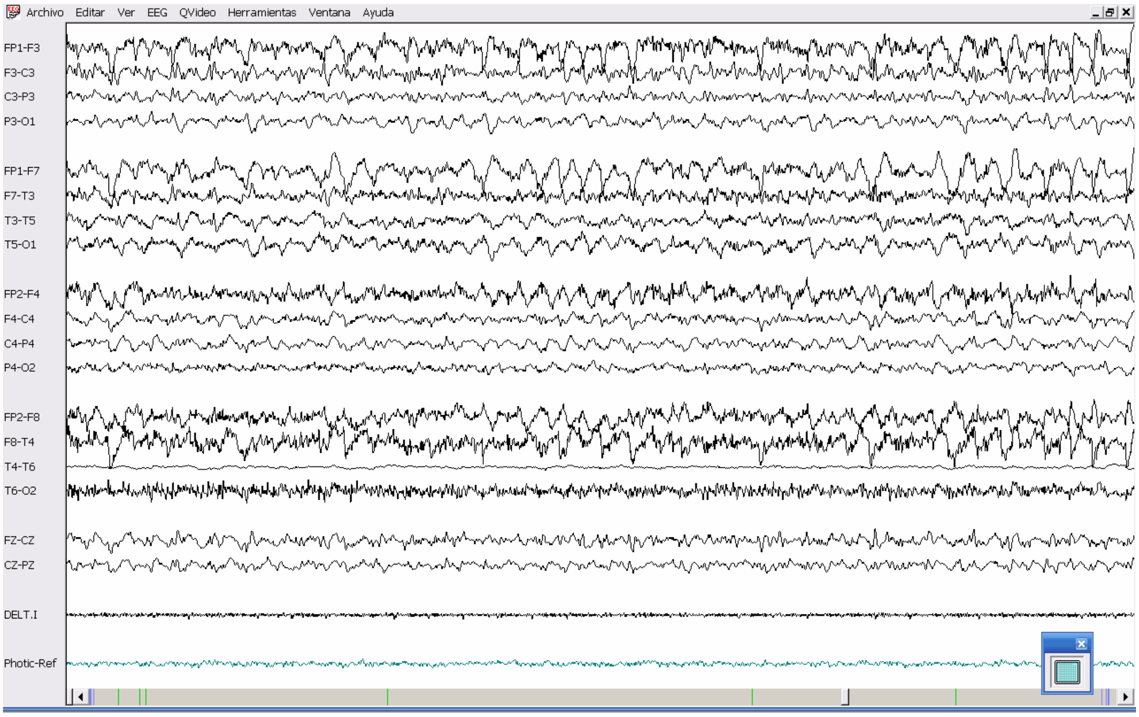
Archivo Editar Ver EEG QVideo Herramientas Ventana Ayuda
FP1-F3
F3-C3
C3-P3
P3-O1
FP1-F7
F7-T3
T3-T5
T5-O1
FP2-F4
F4-C4
C4-P4
P4-O2
FP2-F8
F8-T4
T4-T6
T6-O2
FZ-CZ
CZ-PZ
DELT.I
Photic-Ref

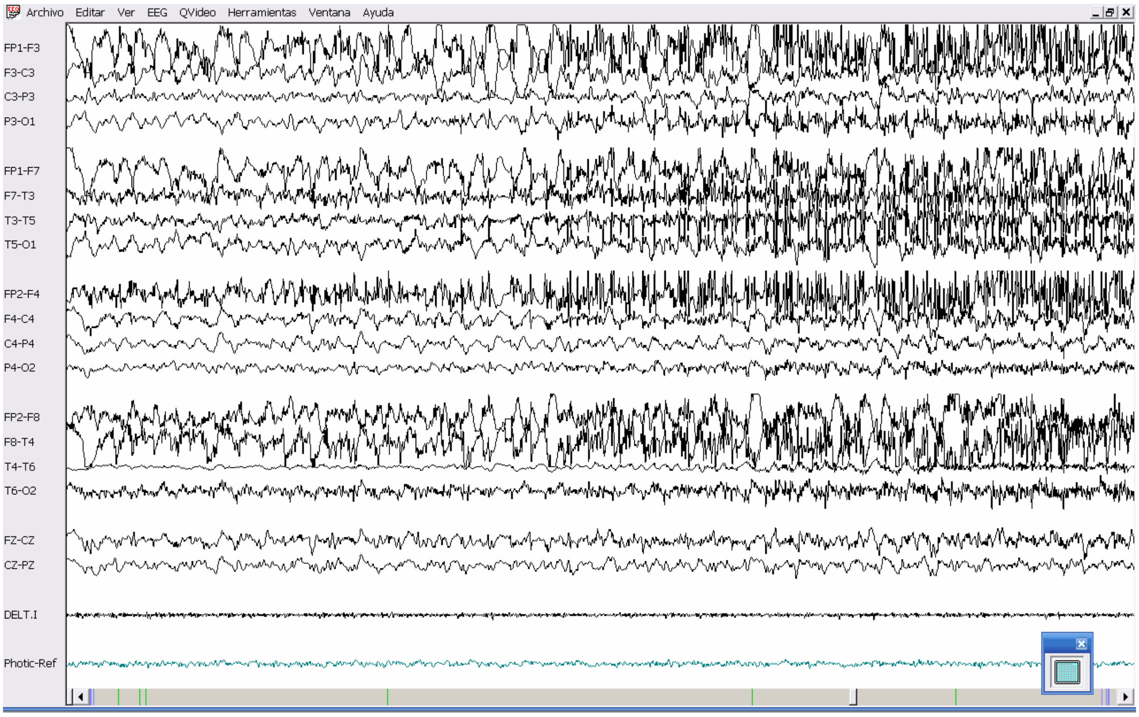
Archivo Editar Ver EEG QVideo Herramientas Ventana Ayuda
FP1-F3
F3-C3
C3-P3
P3-O1
FP1-F7
F7-T3
T3-T5
T5-O1
FP2-F4
F4-C4
C4-P4
P4-O2
FP2-F8
F8-T4
T4-T6
T6-O2
FZ-CZ
CZ-PZ
DELT.I
Photic-Ref

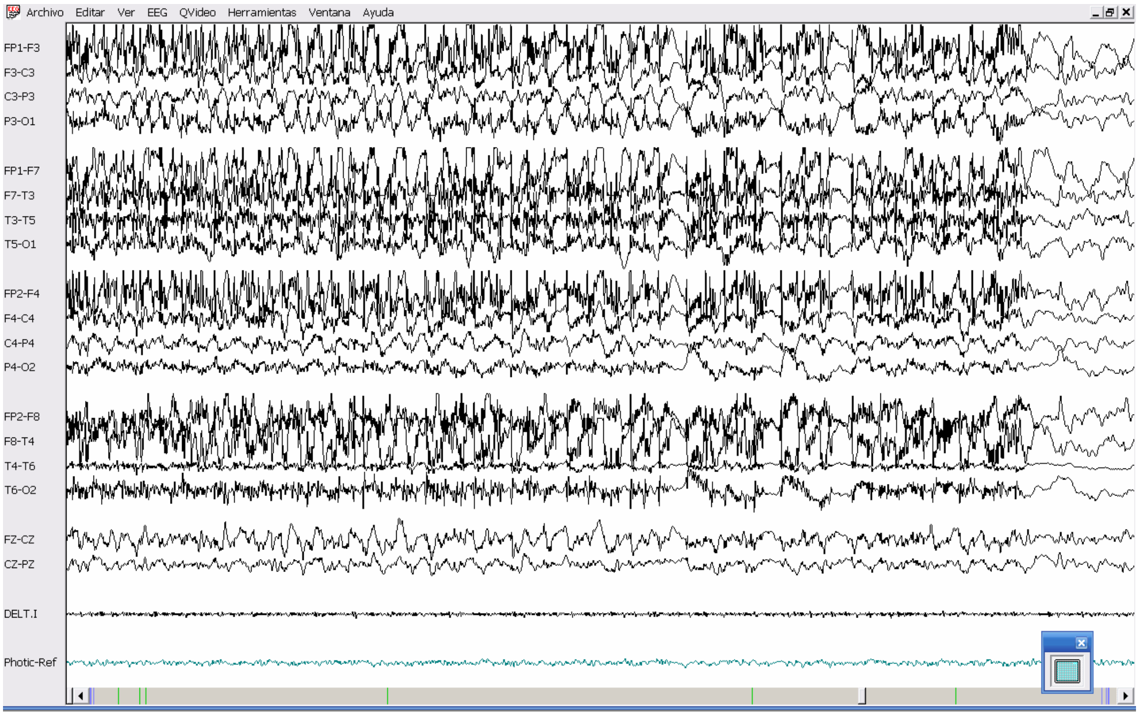
Archivo Editar Ver EEG QVideo Herramientas Ventana Ayuda
FP1-F3
F3-C3
C3-P3
P3-O1
FP1-F7
F7-T3
T3-T5
T5-O1
FP2-F4
F4-C4
C4-P4
P4-O2
FP2-F8
F8-T4
T4-T6
T6-O2
FZ-CZ
CZ-PZ
DELT.I
Photic-Ref

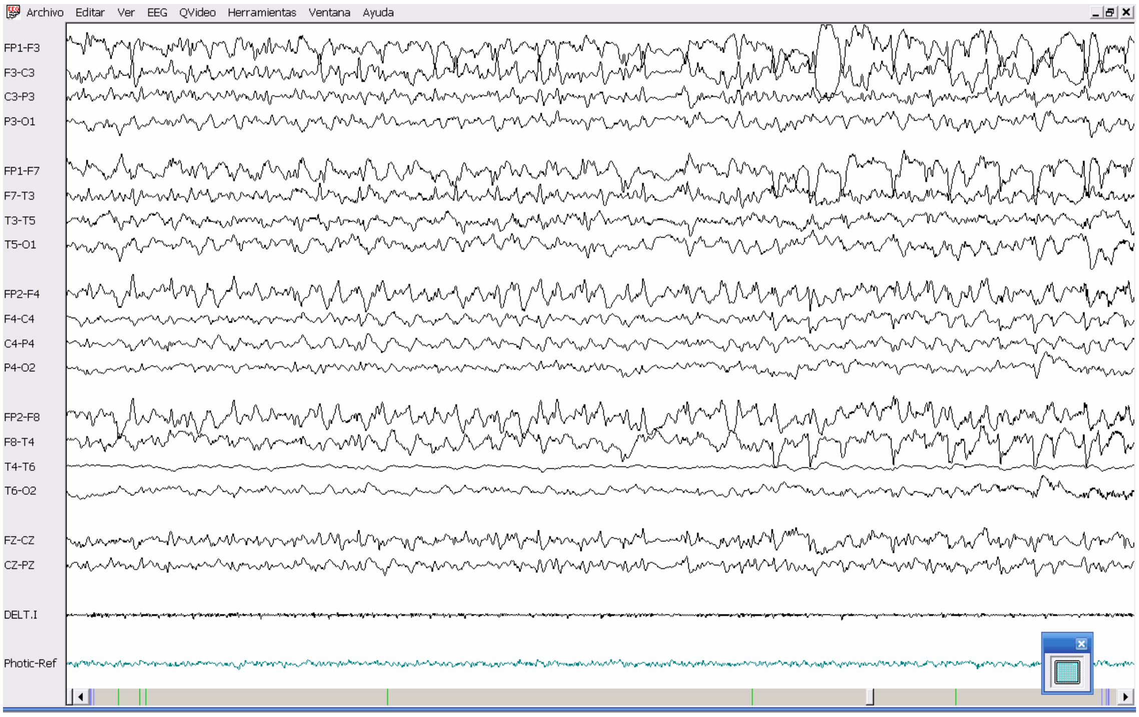

3rd EEG recording at 30 minutes showing similar changes. (15 mm = 1 sec; A = 50 mv)
